# Intramuscular Bleeding and Formation of Microthrombi during Skeletal Muscle Damage Caused by a Snake Venom Metalloprotease and a Cardiotoxin

**DOI:** 10.3390/toxins15090530

**Published:** 2023-08-28

**Authors:** Medha Sonavane, José R. Almeida, Elanchezhian Rajan, Harry F. Williams, Felix Townsend, Elizabeth Cornish, Robert D. Mitchell, Ketan Patel, Sakthivel Vaiyapuri

**Affiliations:** 1 School of Pharmacy, University of Reading, Reading RG6 6UB, UK; m.sonavane@pgr.reading.ac.uk (M.S.); j.r.dealmeida@reading.ac.uk (J.R.A.); e.rajan@reading.ac.uk (E.R.); 2 Toxiven Biotech Private Limited, Coimbatore 641042, Tamil Nadu, India; harryfonsecawilliams@gmail.com; 3 School of Biological Sciences, University of Reading, Reading RG6 6UB, UK; fet7@aber.ac.uk (F.T.); ecornish352@hotmail.co.uk (E.C.); ketan.patel@reading.ac.uk (K.P.); 4 Micregen Ltd., Thames Valley Science Park, Reading RG2 9LH, UK; robertmitchell@micregen.com

**Keywords:** bleeding, cardiotoxin, metalloprotease, muscle damage, thrombosis, microthrombi

## Abstract

The interactions between specific snake venom toxins and muscle constituents are the major cause of severe muscle damage that often result in amputations and subsequent socioeconomic ramifications for snakebite victims and/or their families. Therefore, improving our understanding of venom-induced muscle damage and determining the underlying mechanisms of muscle degeneration/regeneration following snakebites is critical to developing better strategies to tackle this issue. Here, we analysed intramuscular bleeding and thrombosis in muscle injuries induced by two different snake venom toxins (CAMP—*Crotalus atrox* metalloprotease (a PIII metalloprotease from the venom of this snake) and a three-finger toxin (CTX, a cardiotoxin from the venom of *Naja pallida*)). Classically, these toxins represent diverse scenarios characterised by persistent muscle damage (CAMP) and successful regeneration (CTX) following acute damage, as normally observed in envenomation by most vipers and some elapid snakes of Asian, Australasian, and African origin, respectively. Our immunohistochemical analysis confirmed that both CAMP and CTX induced extensive muscle destruction on day 5, although the effects of CTX were reversed over time. We identified the presence of fibrinogen and P-selectin exposure inside the damaged muscle sections, suggesting signs of bleeding and the formation of platelet aggregates/microthrombi in tissues, respectively. Intriguingly, CAMP causes integrin shedding but does not affect any blood clotting parameters, whereas CTX significantly extends the clotting time and has no impact on integrin shedding. The rates of fibrinogen clearance and reduction in microthrombi were greater in CTX-treated muscle compared to CAMP-treated muscle. Together, these findings reveal novel aspects of venom-induced muscle damage and highlight the relevance of haemostatic events such as bleeding and thrombosis for muscle regeneration and provide useful mechanistic insights for developing better therapeutic interventions.

## 1. Introduction

Snakebite envenoming (SBE) is one of the leading causes of mortality and morbidity among rural agricultural communities in many tropical countries [1,2]. SBE has been classified as a high-priority neglected tropical disease by the World Health Organisation (WHO) and causes around 150,000 deaths and 500,000 permanent disabilities worldwide every year [3]. Venom-induced skeletal muscle damage is a key factor of SBE-induced permanent disabilities [4]. Antivenoms are generally not beneficial in treating and preventing SBE-induced muscle damage as the large immunoglobulin molecules are unable to penetrate the damaged local tissues [5,6]. Moreover, the damaged blood vessels and blood clots (thrombi) in capillaries will restrict the blood flow to the affected tissues resulting in ischaemia, further preventing the antivenom from reaching the damaged regions [7]. Extensive tissue damage can necessitate surgical procedures such as fasciotomy (to release the compartment pressure), debridement (to remove the affected tissues), and amputation (to completely remove the affected region/limb to prevent further damage and infection) to manage this condition [8]. Hence, improving our fundamental understanding of how venom toxins affect skeletal muscle and induce permanent muscle damage is critical for developing effective treatments for SBE-induced muscle damage.

Venoms include both enzymatic and non-enzymatic proteins and small peptides [9,10]. Specific venom toxins such as three-finger toxins (3FTX), phospholipase A_2_ (PLA_2_), and snake venom metalloproteases (SVMP) are the main components responsible for causing local tissue damage [7]. An earlier study from our group reported the mechanisms of action involved in skeletal muscle damage induced by a P-III SVMP (named CAMP) from the venom of *Crotalus atrox* in comparison to a 3FTX (cardiotoxin, CTX) from the venom of *Naja pallida* [11]. CAMP induced extensive damage to the extracellular matrix (ECM) and affected the functions of satellite cells and angiogenesis, impairing muscle regeneration. In contrast, CTX caused muscle necrosis although it did not affect satellite cells or the ECM, with full recovery from the damage being achieved through the innate muscle regeneration process [11]. However, the ability of these venom toxins to induce bleeding and thrombosis while causing muscle damage was not compared simultaneously in the previous study. The circulatory system and a continuous blood supply play a vital role in tissue repair and muscle regeneration [12]. However, damage to vasculature results in excessive bleeding, subsequent thrombus formation and inflammatory responses in the affected muscle [7,13,14]. Moreover, thrombus formation will rapidly consume circulating platelets and/or coagulation factors leading to venom-induced consumption coagulopathy, which further augments bleeding [15,16]. Therefore, it is critical to establish the mechanisms of action of muscle-damaging enzymatic and non-enzymatic venom components in inducing intramuscular bleeding and thrombosis. In this study, we determined the ability of CAMP and CTX in inducing bleeding and microthrombus formation in locally damaged skeletal muscle. The outcomes of this study provide evidence to demonstrate the impact of enzymatic (CAMP) and non-enzymatic (CTX) venom toxins in inducing bleeding and thrombosis in muscle tissues.

## 2. Results

### 2.1. CAMP and CTX Induce Damage to the Tibialis Anterior (TA) Muscle in Mice 

**Figure 1:**
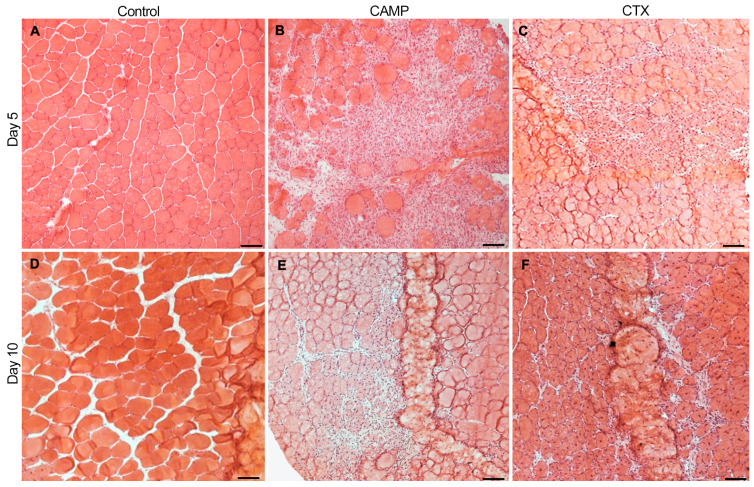
H &E staining of TA muscles treated with CAMP or CTX on days 5 and 10. The TA muscles of mice that were treated with PBS (**A,D**), CAMP (**B,E**) and CTX (**C,F**) were collected on days 5 and 10 following the injection of toxins, and their sections were analysed via H&E staining. The thickness of the muscle sections was 13 μm. The scale bar represents 100 µm. The images shown are representative of experiments performed using five mice in each cohort.

To determine the impact of CAMP and CTX in inducing bleeding and thrombosis in skeletal muscle, they were intramuscularly injected (1 µg of CAMP or CTX in 30 µL of phosphate-buffered saline (PBS)) into the TA muscles of mice. The muscles were collected on days 5 and 10 and used for further analysis. The histological analysis of the muscles using haematoxylin and eosin (H&E) staining confirmed the quality of muscle sections. The undamaged control muscle (injected with the same volume of PBS) displayed a normal morphology (Figure 1A), whereas CTX and CAMP-treated muscles showed clear signs of damage (Figure 1B,C). The infiltration of immune cells was evident in the damaged areas of the muscles treated with CTX and CAMP on day 5. However, on day 10, the muscles showed signs of recovery (the presence of centrally located nuclei in myofibres with reduced spaces between them, a reduced number of infiltrated immune cells and clusters of small fibres), especially in CTX-damaged tissues. These data confirm that both CTX and CAMP damaged the TA muscle as previously reported [11].

### 2.2. CAMP and CTX Induce Bleeding in Damaged Muscle

**Figure 2:**
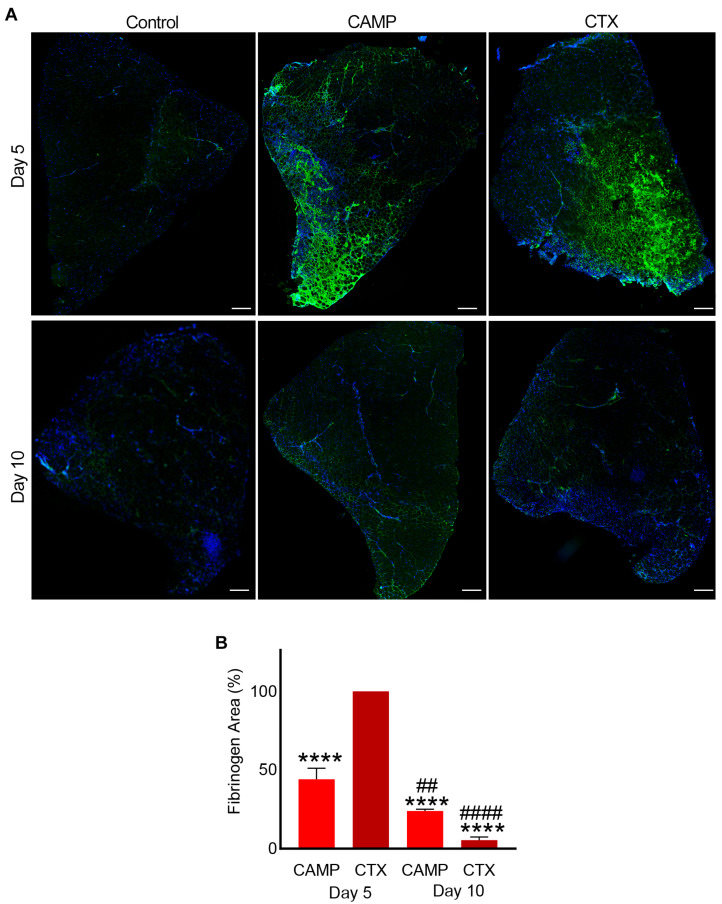
Intramuscular bleeding in muscles treated with CAMP and CTX. FITC-labelled anti-fibrinogen antibodies were used to stain CAMP- and CTX-treated muscle sections along with controls and analyse the extent of bleeding in the muscle at days 5 and 10 post-injection of the toxins. DAPI was used to stain the nuclei. (**A**) Representative images of control, CAMP- and CTX-treated TA muscles at days 5 and 10. (**B**) A bar diagram showing the level of fluorescence at days 5 and 10 in CAMP- and CTX-treated muscles, and their comparisons. The percentage of the fibrinogen area was calculated by dividing the fibrinogen-stained area by the total muscle area. The columns represent mean ± SD (n = 5 mice for each cohort, five sections per mouse). **** *p* < 0.0001 when compared to the level of fluorescence obtained at day 5 in CTX-treated muscle, which was taken as 100% to calculate the relative differences in others. ## *p* < 0.01 and #### *p* < 0.0001 when comparing CAMP- and CTX-treated muscle at day 10 with their corresponding values at day 5. Student’s t-test was used for independent variables. The scale bars represent 100 µm.

Fibrinogen is a highly abundant clotting protein in the blood, and it acts as a scaffold for platelet aggregation and thrombus formation. Therefore, the presence of fibrinogen was measured as a marker of bleeding in the muscle sections. CTX- and CAMP-treated muscle sections on days 5 and 10 along with control muscles were stained using FITC-labelled anti-fibrinogen antibodies. The control muscle showed no fibrinogen, indicating that there was no bleeding in the undamaged tissues (Figure 2A). However, CAMP-treated muscle sections showed the presence of fibrinogen on day 5 (with around 25% fluorescence intensity on average) although it was significantly reduced by day 10 to roughly 12% (Figure 2B). Similarly, the administration of CTX induced bleeding in the muscle on day 5, with a significant reduction on day 10. The percentage of fibrinogen was largely reduced by day 10 compared to day 5 in CTX-treated sections. Although CTX-treated muscle presented significantly higher fibrinogen in the muscle sections than CAMP-treated muscle on day 5, the clearance rate was greater with a reduction from an initial fluorescence intensity of 50% to 3% by day 10 in CTX-treated muscle. The presence of fibrinogen in both toxin-treated muscles indicated intramuscular bleeding, although we cannot rule out the staining of thrombi as they also contain fibrinogen.

### 2.3. Microthrombus Formation in CTX- and CAMP-Damaged Muscles

Microthrombi are small blood clots or aggregates of platelets and fibrin formed in capillaries and/or tissues. Upon stimulation of platelets, the inside-out signalling to the integrin αIIbβ3 switches its conformation from a low-affinity state to a high-affinity state for fibrinogen binding, which causes the aggregation of platelets via the use of fibrinogen as a scaffold [17]. Similarly, P-selectin is secreted from α-granules upon platelet activation. Both of these factors play a critical role in the formation of platelet-mediated blood clots [17]. Therefore, the presence of integrin αIIbβ3 and P-selectin exposure on the surface of platelets confirms the existence of platelet aggregates/thrombi. The muscle sections obtained from CTX- and CAMP-treated mice were stained with FITC-labelled anti-integrin αIIbβ3 and anti-P-selectin antibodies.

**Figure 3:**
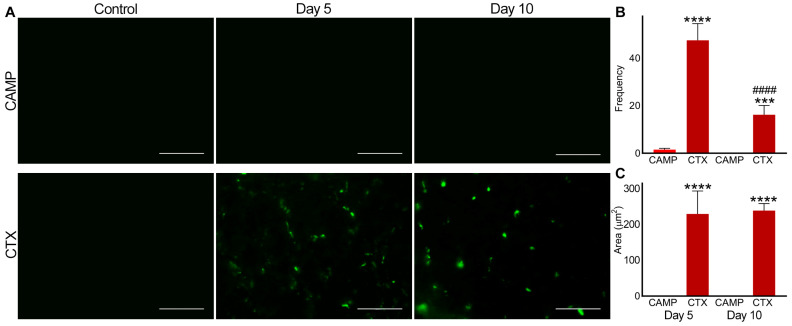
Analysis of microthrombus formation in CAMP- and CTX-damaged muscle by staining integrin αIIbβ3. FITC-conjugated anti-integrin αIIbβ3 antibodies were used to stain and analyse microthrombus formation in TA muscles injected with PBS (control), CAMP or CTX on days 5 and 10 post-injection of the toxins. (**A**) Representative images of experiments performed with five mice in each cohort. Muscle sections were imaged at a 40× magnification. Images were analysed using the ImageJ 3D object counter to obtain the frequency (**B**) and area (**C**) of the microthrombi in CAMP- and CTX-treated muscles. The columns represent the mean ± SD (n = 5 mice for each cohort, five sections per mouse). *** *p* < 0.001 and **** *p* < 0.0001 when comparing CTX with CAMP on the corresponding day. #### *p* < 0.0001 when comparing CTX day 10 to CTX at day 5. A one-way ANOVA followed by Tukey’s test was used to analyse the data. The scale bars represent 50 µm.

In CAMP-treated muscle, there was no detectable level of fluorescence observed for integrin αIIbβ3 on either day 5 or 10 (Figure 3). However, CTX-treated muscle on days 5 and 10 displayed the presence of microthrombi as measured through the level of fluorescence for integrin αIIbβ3. Moreover, in CTX-treated muscles, the frequency of microthrombi was significantly reduced by day 10 compared to that on day 5, although the area of the microthrombi within the muscle remained the same.

**Figure 4:**
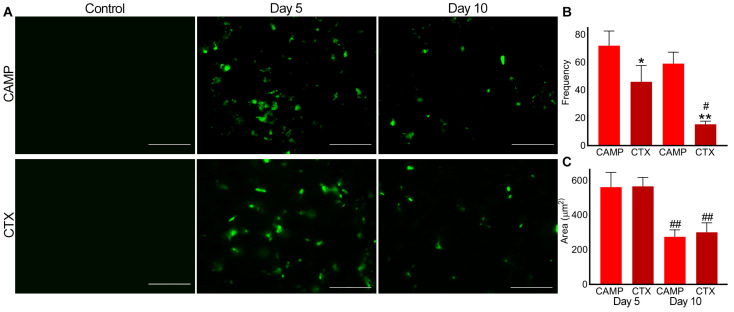
Analysis of microthrombus formation in CAMP- and CTX-induced muscle damage using P-selectin as a marker. FITC-conjugated mouse anti-P-selectin antibodies were used to stain and analyse microthrombus formation in mouse TA muscles injected with PBS (control), CAMP or CTX. (**A**) Representative images of experiments performed with five mice. The muscle sections were imaged at a 40× magnification. Images were analysed using an ImageJ 3D object counter to obtain the frequency (**B**) and area (**C**) of the microthrombi in CAMP- and CTX-treated muscles. The columns represent the mean ± SD (n = 5 mice for each cohort, five sections per mouse). * *p* < 0.05, and ** *p* < 0.01 when compared with CAMP on the corresponding day. # *p* < 0.05 and ## *p* < 0.01 when compared with the same toxin on day 5. A one-way ANOVA followed by Tukey’s test was used to analyse the data. The scale bars represent 50 µm.

Similarly, P-selectin was absent in the control muscle sections (Figure 4). However, it was evident in CAMP- and CTX-treated muscle sections on days 5 and 10, confirming the presence of microthrombi. On day 5, the frequency of microthrombi was around 20% higher in CAMP-treated muscles compared to that in CTX-treated muscles, although there was no difference in the size of the thrombi. Even on day 10, the frequency of the microthrombi was around 40% higher in CAMP-damaged muscles compared to that in CTX-treated muscles. However, CTX-treated muscles showed a significant reduction in microthrombus frequency of around 50% on day 10. The area of microthrombi was reduced by almost 50% on day 10 in both CAMP- and CTX-treated muscles compared to that on day 5.

### 2.4. CTX Extends Clotting Time

**Figure 5:**
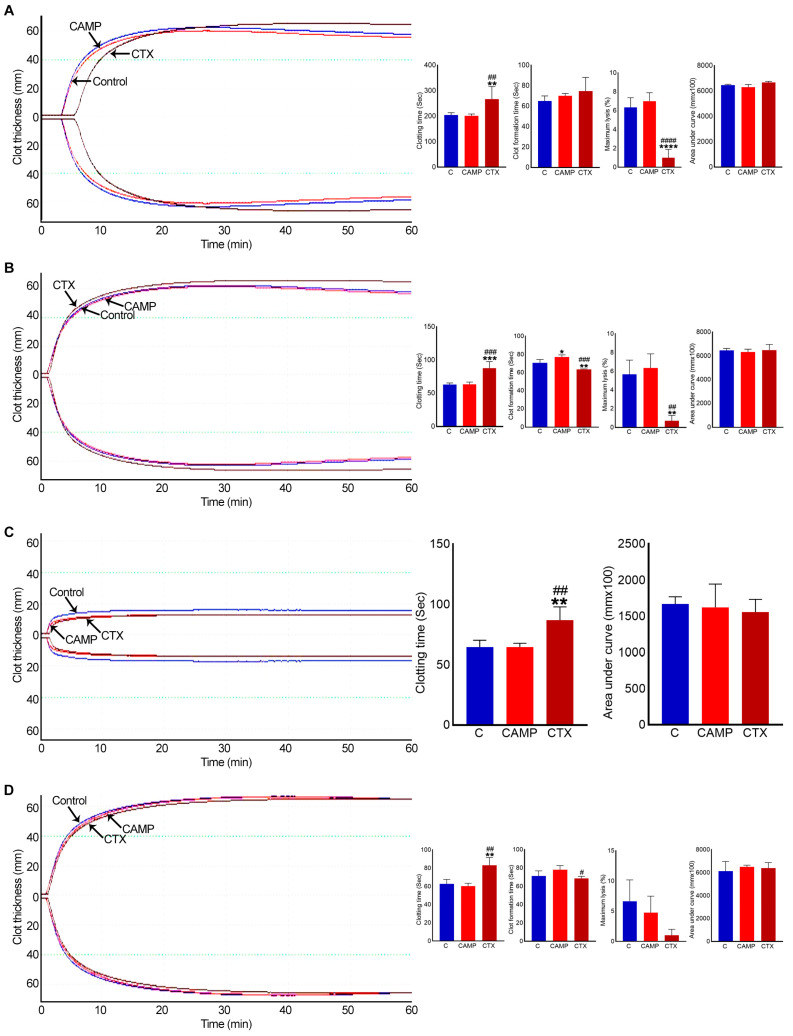
ROTEM analysis in whole human blood with CAMP and CTX. (**A**) Intem, (**B**) extem, (**C**) fibtem and (**D**) aptem data showing the impact of 10 μM CAMP or CTX in human whole blood clotting via different pathways. Graphs represent clotting time, clot formation time, maximum lysis, and area under the curve. The columns represent the mean ± SD (n = 4 independent donors from whom the blood samples were obtained for these experiments). * *p* < 0.05, ** *p* < 0.01, *** *p* < 0.01 and **** *p* < 0.0001 when compared to the control group (**C**). # *p* < 0.05, ## *p* < 0.01, ### *p* < 0.001 and #### *p* < 0.0001 when compared to CAMP. A one-way ANOVA followed by Tukey’s test was used to analyse these data.

To determine the direct effects of CAMP and CTX on blood clotting in human blood under in vitro settings, a rotational thromboelastometry (ROTEM) analysis was performed using human-citrated whole blood. The data of the intem analysis, which evaluates the intrinsic and common pathways, confirmed that clotting was delayed when CTX was added to the whole blood (Figure 5A). Moreover, it reduced fibrinolysis by around 30% compared to the controls. However, there was no significant difference in the clot size (area under the curve) or in clot firmness (data not shown) between the control and CTX-induced clots. Similarly, the extem analysis, which evaluated the extrinsic and common pathways, showed a delay in clotting time and reduced fibrinolysis in CTX-treated blood (Figure 5B). The impact of CTX on clotting, independently of platelets, was determined via fibtem analysis. This assay confirmed that clotting time was delayed, although clot firmness and size remained unchanged (Figure 5C). The aptem analysis (in the absence of fibrinolysis) suggested that CTX delayed blood clotting but slightly accelerated the clot formation time compared to that with CAMP (Figure 5D). These results indicate that CTX affects blood clotting through multiple coagulation pathways independently of platelets and fibrinolysis.

When similar experiments were performed using CAMP, it showed no major changes in clotting time, clot formation time, clot firmness or lysis compared to the controls in any analysis. Only in extem analysis did CAMP delay the clot formation time by around 10%.

## 3. Discussion

The life-threatening pathophysiology of SBE is driven by the individual and synergistic actions of biologically active venom toxins with different molecular targets, which can lead to various effects in the body including haemostatic disturbances and muscle damage [18,19]. At the clinical level, venom-induced consumption coagulopathy (VICC) with diverse manifestations triggered by different toxins is a serious issue, and the resulting haemostatic effects may vary depending on the consumed factor in the coagulation cascade [20,21]. This systemic and potentially lethal phenomenon following SBE in patients is recognised via the activation of the clotting cascade and/or elevated degradation of the fibrinogen [22]. At a molecular level, toxins can affect thrombus formation via varied targets and/or due to their thrombolytic properties, resulting in thrombotic/bleeding complications [14]. However, the coordination and regulation of haemostatic responses following skeletal muscle injury including intramuscular bleeding and thrombosis have not been fully understood. Determining the differences that contribute to these haemostatic events by studying clinically relevant venom toxins is key to elucidating the general impacts on muscle damage and subsequent regeneration. Due to the role that the circulatory system plays in wound healing and tissue regeneration, it is vital to study its state during venom-induced muscle damage. The circulatory system delivers leukocytes to damaged areas to enable the clearing of cell debris and to prevent infections [23]. Additionally, the limited effectiveness of currently used antivenom treatment for managing local tissue injury and mitigating its long-standing consequences [5,24] reiterates the importance of a comprehensive investigation of venom-induced muscle damage including haemostatic elements to allow the development of better management strategies for this condition. Therefore, we analysed a parallel comparison of haemotoxicity induced by enzymatic (CAMP) and non-enzymatic (CTX) muscle-damaging venom components during skeletal muscle damage to establish their diverse effects.

SVMPs are crucial components in viper venom-induced myotoxicity due to their ability to hinder adequate muscle regeneration and complete functional recovery following acute damage [25]. The proteolytic activity of these toxins causes important alterations to different components of the muscle architecture, especially the ECM, which is essential for muscle regeneration [4,11]. CAMP was previously reported to damage the ECM in skeletal muscle [11]. As shown here, during CAMP-induced muscle injury, there was extensive local damage with significant bleeding, as evidenced by fibrinogen in the injured muscles. In vitro studies have previously demonstrated that some P-III SVMPs have a fibrinogenolytic effect on human plasma fibrinogen [26]. Therefore, CAMP might directly cleave plasma fibrinogen as well as affecting the ECM in blood capillaries during muscle damage to induce bleeding. Earlier research has established that viper venoms contain procoagulant toxins that can induce VICC due to the consumption of clotting factors [27]. These proteolytic enzymes often cause rapid clot formation in vitro but can induce bleeding complications due to the rapid consumption of several factors. Fibrinogen, the common point of the clotting pathway, is the most consistently consumed factor in VICC [15]. Notably, some studies have revealed that SVMPs could cleave integrins and fibrin clots, leading to further bleeding [28,29]. As a P-III SVMP, CAMP has a disintegrin-like functional domain in its structure but a possible reason for the lack of the integrin αIIbβ3 signal in the muscle tissues is likely due to its direct effect on this integrin shedding. Integrin αIIbβ3 plays a crucial role in platelet aggregation and adhesion [30]. Therefore, a lack of integrin αIIbβ3 might be one of the main causes of extensive and prolonged bleeding in CAMP-damaged muscle. Treatment with CAMP enhanced the detection of P-selectin in the microthrombi of damaged tissue. CAMP treatment showed a high frequency of P-selectin microthrombi within the muscle even at a later time point, indicating ongoing muscle damage. Moreover, CAMP may possess thrombolytic properties, and the reduction in the microthrombi area could be due to thrombolysis. Interestingly, CAMP exhibited an insignificant effect on intem and extem analysis. The intrinsic pathway is initiated by activators such as collagen, which is a substrate for CAMP. Hence, the lack of collagen in the blood due to CAMP activity could indicate the indirect effects of CAMP on intem analysis. These findings highlight the mechanistic action of a purified toxin, CAMP, on inducing bleeding and thrombotic complications during muscle damage. However, this cannot be generalized to all SVMPs in diverse venoms as they possess variable potencies, substrate specificities and a diverse range of pharmacological properties, which can also interact with other families of toxins to exert synergistic activities. Similarly, when whole venom is used, the level of haemotoxic effects may vary compared to those of the purified toxin.

In the CTX-induced injury model, we observed acute muscle damage accompanied by bleeding and thrombosis. CTX extended the clotting time in ROTEM analysis, which suggests its ability to cause bleeding. The likely mechanisms of CTX-induced bleeding may include the necrosis of endothelial cells in microcapillaries that perfuse the damaged muscle and/or anticoagulant properties of these non-enzymatic molecules. In the first scenario, lysis or necrosis of the cell membrane leads to capillary permeation, causing blood to leak into the interstitial space of muscle tissue. This event could also be related to the activation of native matrix metalloproteases, which regulate vascular ECM and homeostasis [31]. This increases blood flow to the damaged site and aids the perpetuation of the haemorrhagic effect [32,33]. In the second case, some 3FTXs can bind and inhibit specific coagulation factors or complexes [34]. For example, members of this toxin family, such as hemextin A, ringhalexin and exactin have been proposed as potential anticoagulant lead molecules for the development of therapeutics, research tools and diagnostic probes due to their selective effects on specific coagulation factors [35,36]. The first wave of haemostasis is due to the accumulation of platelets at the site of the injury [37]. Platelet activation and thrombus formation are achieved by the modulation and binding of various receptors on the platelet surface to their ligands. Platelet integrins and their ligands initiate stable adhesion, and inside-out signalling to integrin αIIbβ3 recruits more platelets for aggregation. The integrin αIIbβ3 enhances platelet activation through cytoskeleton rearrangement and granule secretion, thereby facilitating haemostatic plug or thrombus formation. The lack of integrin αIIbβ3 reduces platelet aggregation and impairs thrombus growth. In CTX-injured muscle sections, integrin staining revealed clots, which may be one of the reasons why CTX-induced bleeding gradually improves. P-selectin plays a crucial role in thrombus formation and wound healing pathways by recruiting white blood cells to the injured site. The frequency of P-selectin-stained thrombi decreased in CTX-mediated damage with the progression of tissue repair, suggesting that the muscle damage was advancing towards a resolution. Similar results were reported in a recent study exploring the contribution of platelet-released chemokines to successful muscle regeneration [38]. In this study, platelet thrombi were monitored using anti-GP1bβ antibodies, which showed the presence of these aggregates in early stages (on days 1 and 7), with a considerable reduction at day 14 in the CTX-induced muscle injury model [38]. Our CTX-induced muscle injury model corroborated these previous findings. Additionally, the same study showed how platelet-derived signals modulate neutrophil recruitment and coordinate a favourable niche for immune infiltration and myogenesis that precede the restoration of muscle structure and function [38]. The use of neutrophil-depleted mice has revealed the active involvement of neutrophils in the regenerative phase following the damage caused by *Bothrops asper* [39]. Imbalances in these events can establish a persistent inflammatory state with a predominance of atrophic mediators that culminate in unresolved damage, as observed in the CAMP-induced muscle injury model. The ROTEM analysis showed that CTX may affect intrinsic and extrinsic pathways as well as the common pathway as it delayed clotting time in all analyses. This suggests the need for further investigations to establish the exact contribution of CTX to clotting cascades and thereby, intramuscular bleeding and thrombosis.

**Figure 6:**
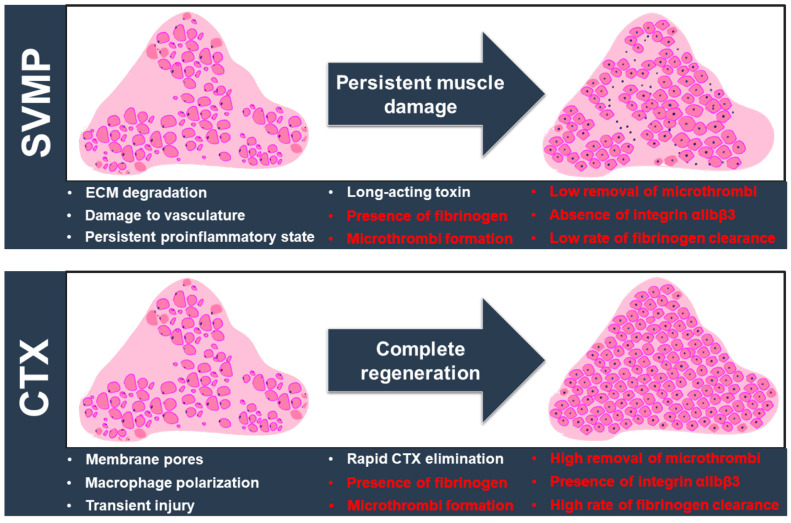
The landscape of venom-induced muscle damage by SVMPs and CTX. SVMP (specifically CAMP) and CTX induce extensive muscle damage through different actions (as shown in white), but their different mechanisms of action lead to divergent outcomes. Key factors influencing the muscle regeneration process are highlighted here. The different outcomes developed following SVMP/CTX-induced muscle damage are shown in red. SVMP hydrolyses the ECM components and causes vascular alterations, with a significant impact on the influx of inflammatory cells. A persistent proinflammatory environment drives the excessive deposition of fibrous connective tissues with alterations in muscle architecture and functional implications. On the other hand, CTX triggers an acute, transient injury through a membranolytic action without compromising the ECM and vasculature. A spatiotemporal transition of inflammatory cells promotes a myogenic program resulting in complete regeneration. The normal architecture of the muscle is then re-established with a homogenous distribution of fibre sizes and morphology. We evidenced the intramuscular presence of fibrinogen and microthrombus formation in muscle damage induced by both toxins. However, significant differences in terms of the clearance rate of fibrinogen, removal of microthrombi and levels of integrin αIIbβ3 were detected. They may be related to the two contrasting muscle regeneration profiles. Another important aspect to be considered is the kinetic action and removal of myotoxins. The long-term presence of SVMPs is likely to drive repetitive degradative/regenerative cycles that hamper the complete elimination of fibrinogen and microthrombi and exacerbate the inflammatory state that impairs tissue repair. In contrast, CTX has a transient activity that culminates in the successful removal of fibrinogen and microthrombi, which should result in a favourable outcome for regeneration.

The paradigm of successful or poor muscle regeneration following elapid and viper envenomation has been mainly discussed with relevance to the impact of the protagonist toxins and their effects on essential components for myogenesis, such as the ECM, inflammation, and blood supply [11,40,41]. Figure 6 summarises the current knowledge of venom toxin-mediated muscle damage with key features/events that affect the regenerative process. The results of this study are also included in this scheme and highlighted in red. Earlier studies have shown that CAMP damages the capillaries and hinders angiogenesis in damaged muscles [42,43]. On the other hand, the degradation of the ECM in skeletal muscle leads to a lack of scaffolding for myogenesis and angiogenesis [44]. Hence, muscle tissue struggles with regeneration when P-III metalloproteases are administered. In a previous study from our group, CTX caused no change in the number of capillaries per muscle fibre [11]. This observation suggested that CTX does not have direct haemotoxic properties through affecting ECM and angiogenesis. The current results based on different biomarkers show that haemorrhage occurs in CTX-damaged muscle. The actions of matrix metalloproteases in vasculature remodelling and tissue regeneration after muscle injury may also contribute to bleeding and subsequent thrombosis [45]. Interestingly, the level of fibrinogen was significantly reduced with the progression of muscle regeneration. This reduction in fibrinogen aligns with the fact that intact blood capillaries facilitated muscle repair. Since fibrinogen deposition can promote a profibrotic environment, the reduction in fibrinogen levels in CTX-induced muscle injury is consistent with reduced fibrosis and better functional outcomes during elapid snake envenomation [46]. However, a lower clearance rate was detected in CAMP-damaged muscle sections. As discussed earlier and supported by previous studies [47,48,49], neutrophils are fundamental players in muscle repair progress and they help to orchestrate a pro-reparative scenario dictated by macrophage phenotype transition [50]. Neutrophil recruitment to damaged tissue in turn can be modulated by platelet-derived chemokines and microthrombus formation, which may account for differences in the removal rates of fibrinogen and intramuscular microthrombi [38,51]. The action and temporal presence of these myotoxins must also be considered in this speculative view. Previous studies have shown the long-lasting presence of SVMP, which leads to continuous ECM degradation and haemorrhagic effects [11,52]. The temporal action of cardiotoxin is in line with the restoration of muscle architecture and complete removal of the signs of intramuscular bleeding [53,54]. In summary, the accumulation of this evidence supports the current concept of poor muscle regeneration in viperid envenomation due to direct damage to vasculature with dysregulated responses, subsequent poor repair, and severe tissue destruction [55]. As a result, this reduced remodelling in the CAMP-induced muscle injury model leads to extensive collagen deposition, seen as the excessive muscle accumulation of fibrous connective tissue that affects the motile and contractile functions of SBE victims [56].

In summary, this comparative study demonstrates intramuscular bleeding and microthrombus formation following SVMP- or 3FTX-induced myotoxicity. Overall, it offers valuable insights into the homeostasis of muscle tissue and their rearrangements induced by catalytically active and non-enzymatic venom molecules with their specific actions on haemostatic processes. This highlights the importance of studying disturbances induced by overlooked toxins that may contribute to the severe pathological sequelae observed in skeletal muscle. Our observational study raises a series of hypotheses that deserve deeper analysis. For example, the presence of fibrinogen and potential intramuscular bleeding in CTX-induced muscle injury needs further investigation. Additionally, research focused on pharmacological interventions is important to identify the extent and the role of these alterations and the underlying mechanisms that impede muscle regeneration. By integrating their mechanism of action in haemostatic effects, we provide a better picture of the toxins’ impacts on thrombus formation, bleeding, and vascular damage in venom-induced muscle injury. These will pave the way to a comprehensive understanding of the processes involved in muscle damage and its possible implications for the development of life-changing solutions to treat venom-induced muscle injury, facilitate tissue regeneration, and prevent long-term physical sequelae. In practical terms, the multidimensional nature of muscle damage and the diversity of underlying factors require more detailed investigations that may culminate in therapeutic benefits and clinical translation. Future studies should explore whether or not variations in toxins would influence the pattern of haemostatic responses during muscle damage described here and evaluate the neutralisation of these muscle-perturbing venom proteins using current and next-generation antivenom therapies. 

## 4. Materials and Methods

### 4.1. Materials Used

Purified CTX from the venom of the red-spitting cobra (*Naja pallida*) was purchased from Latoxan (Valence, France). Lyophilised *Crotalus atrox* (*C. atrox*) venom was purchased from Sigma Aldrich (Gillingham, UK). CAMP was purified from the venom of *C. atrox* using a combination of ion exchange and gel filtration chromatography, as reported previously [11].

### 4.2. Injection of Venom Toxins in TA Muscles of Mice

All mice were anaesthetised using 3.5% (*v*/*v*) isoflurane in oxygen and then kept at 2% (*v*/*v*) isoflurane throughout the procedure. An amount of 1 µg of purified CAMP or CTX in a 30 µL volume was injected into the left TA muscle. The right TA muscle was given a control injection of 30 μL of PBS. The mice were monitored for either 5 or 10 days before being sacrificed via CO_2_ inhalation and muscle collection.

### 4.3. Dissection and Processing of Tissues

The tissue samples were collected on either day 5 or 10 following the injection of toxins. Animals were dissected, and the TA muscles were carefully removed from the tendon to avoid mechanical damage. The muscle samples were frozen in liquid nitrogen-cooled isopentane and kept at −80 °C. The muscle tissue was then embedded in Tissue-TEK^®^ OCT (Optimal Cutting Temperature) medium and sliced into 13 μm thick transverse sections using a cryo-microtome. These sections were placed onto glass slides and stored at −80 °C until required for further use.

### 4.4. H&E Staining of Muscle Sections

The muscle sections on glass slides were removed from the −80 °C freezer and left at room temperature for 15 min, and later, the sections were soaked in PBS to rehydrate them. The slides were then submerged in Harris haematoxylin stain for two minutes. After rinsing the slides in water for two minutes, they were dipped twice in 70% acidic alcohol (70% ethanol (*v*/*v*) and 0.1% (*v*/*v*) HCl) and then rinsed again in water for five minutes. The slides were then placed into a container with a 1% (*w*/*v*) eosin solution for two minutes and then transferred into a slide container containing 70% ethanol. The slides were dehydrated by soaking them in 70%, 90%, and 100% ethanol. Finally, the slides were transferred into xylene for two rounds of three minutes. The sections were fixed using distyrene, plasticiser and xylene (DPX) mounting media. The muscle sections were observed and imaged using a Zeiss AxioImager light microscope (five sections per mouse, 5 mice for each cohort).

### 4.5. Immunohistochemistry of TA Muscle Sections

Using a wash buffer solution (PBS with 5% (*v*/*v*) foetal bovine serum and 0.05% (*v*/*v*) Triton X-100), FITC-conjugated primary antibodies (anti-human fibrinogen antibodies from Agilent Technologies, Stockport, UK and anti-integrin αIIbβ3 and anti-P-selectin antibodies were from Emfret Analytics, Eibelstadt, Germany) were diluted at 1:50 dilution. The slides were cleaned and hydrated three times with PBS for 5 min each. Next, a permeabilisation buffer (20 mM HEPES, 3 mM MgCl_2_, 50 mM NaCl, 0.05% (*w*/*v*) sodium azide, 300 mM sucrose and 0.5% (*v*/*v*) Triton X-100) was added and allowed to incubate for 15 min. A blocking wash buffer was added and incubated for 30 min. The pre-made primary antibodies were added, and the slides were incubated for 1 h at room temperature. Unbound antibodies were washed off, and the slides were mounted in 6-diamino-2-phynolinodole (DAPI) containing mounting media. The sections (five sections per mouse and five mice for each cohort) were visualised, and the images were obtained using a Zeiss AxioImager fluorescence microscope (Zeiss Microscopy Ltd., Cambridge, UK). For fibrinogen, the whole-muscle image which was made using multiple images of that muscle at a 10× objective, was analysed using threshold analysis. The baseline threshold was set using the muscle image of an undamaged mouse. For P-selectin and integrin αIIbβ3, multiple images for each muscle section from each mouse were taken using a 40× objective. Using the Image J (version 1.53k, NIH, Bethesda, MD, USA) 3D analysis tool, the baselines were set using undamaged contralateral muscle and then the number and area of thrombi were quantified. For each mouse, the values were calculated as means and then analysed together with the data obtained from all animals.

### 4.6. ROTEM Analysis

The ROTEM Delta analyser (Werfen, UK), was used to study the effects of CAMP and CTX on human whole blood clotting. Intem and extem analyses were conducted to determine the impact of venom toxins on intrinsic and extrinsic as well as common pathways of blood clotting, respectively. Fibtem analysis was carried out to determine the effects of toxins on clotting in the absence of platelets, while aptem analysis was completed to assess the impact of venom toxins on blood clotting in the lack of fibrinolysis. In each assay, 10 µM of CAMP or CTX was mixed with 300 μL of citrated whole human blood and pre-set volumes of the respective reagents for different assays in accordance with the manufacturer’s instructions. The blood samples were recalcified using a startem reagent (0.2 M CaCl_2_ in a HEPES buffer, pH 7.4), and clotting was initiated using intrinsic (partial thromboplastin phospholipid from rabbit brain and ellagic acid) and extrinsic clotting activators (recombinant tissue factor, phospholipids, and heparin). The fibtem (cytochalasin D and 0.2 M CaCl_2_ in the HEPES buffer, pH 7.4) and aptem (aprotinin and 0.2 M CaCl_2_ in the HEPES buffer, pH 7.4) assays were performed using specific reagents before the initiation of clotting using the extem activation reagent. Various parameters of whole blood coagulation were analysed using ROTEM assays.

### 4.7. Statistical Analysis

All statistical analyses were performed using GraphPad Prism 8. Based on the data type, a student t-test or one-way ANOVA was used to calculate the *p* values to determine statistical significance. All the staining procedures and analyses were performed blindly by individuals who were not involved in experimental procedures on mice.

## Data Availability

All data from this study are included within this manuscript.
